# TRIM14 Overexpression Induces Chemoresistance and Malignant Behaviors of Hepatocellular Carcinoma Cells by Activating the STAT3/HIF-1α Pathway

**DOI:** 10.3390/ijms241612589

**Published:** 2023-08-09

**Authors:** Weiqi Xu, Lihong Zhuang, Hongxu Zhu, Anrong Mao, Jiamin Zhou, Lu Wang

**Affiliations:** 1 Department of Hepatic Surgery, Shanghai Cancer Center, Fudan University, Shanghai 200032, China; 2 Department of Oncology, Shanghai Medical College, Fudan University, Shanghai 200032, China; 3 Qingdao Institute, Department of Hepatic Surgery, Fudan University Shanghai Cancer Center, Qingdao 266500, China

**Keywords:** TRIM14, hepatocellular carcinoma, chemoresistance, pathway, STAT3

## Abstract

Members of the tripartite motif (TRIM)-containing protein family have been found to be involved in the progression of hepatocellular carcinoma (HCC). TRIM14 exerts a promotive impact on several cancers. This study aimed to explore the function and mechanism of TRIM14 in HCC. TRIM14 expression in HCC tissues and HCC cell lines was detected. The overexpression or knockdown model of TRIM14 was established in HCC cell lines. Cell Counting Kit-8 (CCK-8) assay, flow cytometry, Transwell assay, RT-PCR, Western blot, and immunofluorescence were performed to verify the influence of TRIM14 on cell proliferation, sensitivity to chemotherapy drugs, apoptosis, migration, invasion, and autophagy. A xenograft tumor model was used to confirm the impact of TRIM14 on tumor cell growth. As shown by the data, TRIM14 level was notably higher in the tumor tissues of HCC patients than in the adjacent tissues. The overall survival rate of patients with a high TRIM14 expression was relatively lower than that of patients with a low TRIM14 expression. TRIM14 upregulation enhanced the proliferation, autophagy, migration, and invasion of HCC cells and chemoresistant HCC cells and decreased apoptosis. TRIM14 knockdown contributed to the opposite effects. In in vivo experiments, TRIM14 upregulation bolstered tumor growth. Western blot analysis revealed that TRIM14 upregulation boosted signal transducer and activator of transcription3 (STAT3) and hypoxia-inducible factor-1alpha (HIF-1α) expression, and TRIM14 knockdown suppressed their expression. Moreover, repressing STAT3 and HIF-1α could mitigate the tumor-promoting role of TRIM14 in HCC cells. Overall, TRIM14 facilitated malignant HCC development and induced chemoresistance in HCC cells by activating the STAT3/HIF-1α axis.

## 1. Introduction

Liver cancer, the most ubiquitous malignant tumor with fatality worldwide, is categorized as primary liver cancer and metastatic liver cancer [1]. Of all patients suffering from liver cancer, over 90% are afflicted by hepatocellular carcinoma (HCC). Generally, patients have already reached the advanced stage of cancer once they are diagnosed, accompanied by an exceptionally poor prognosis [2]. The primary therapies for liver cancer include radiofrequency ablation, hepatectomy (HR), liver transplantation, and targeted treatment with transcatheter arterial chemoembolization (TACE). Great challenges remain, especially in Asia [3]. HCC cells can easily develop resistance to chemotherapy drugs, reducing the effects of liver cancer treatment [4]. Therefore, new remedies for HCC are warranted.

TRIM proteins are a multifunctional family of ubiquitin E3 ligases consisting of over 70 members and displaying critical functions in the immune response and carcinogenesis. For instance, TRIM6 can boost rectal cancer cell proliferation [5]. TRIM29 expression pertains to the poor prognosis of gastric cancer and cervical cancer [6]. TRIM14, situated at 9q22.33 and 4480 bp in length, is involved in multiple cell processes—including intracellular signal transduction, growth, and apoptosis—and is related to the pathogenesis of miscellaneous cancers [7]. For instance, TRIM14 overexpression in vivo substantially amplifies the malignancy of tongue squamous cell carcinoma and ex vivo strengthens its resistance to cisplatin [8]. TRIM14 overexpression can also boost osteosarcoma cell proliferation, metastasis, and invasion in vitro and enhance tumor growth in vivo, but TRIM14 knockdown produced the reverse outcome [9]. Nevertheless, the molecular mechanism of TRIM14 in HCC cells needs further exploration.

The signal transducer and activator of transcription 3 (*STAT3*), a transcription factor of tyrosine phosphorylation located at 17q21.2 with a length of 4899 bp, can be activated by cytokines and nonreceptor tyrosine kinases, participating in cancer cell proliferation, metastasis, and invasion [10,11]. STAT3 pathway activation can bolster HCC cell growth and invasion [12]. *HIF-1A*, situated at 14q23.2 and 3865 bp in length, is a subtype of hypoxia-inducible factors that participates in cell proliferation, differentiation, and apoptosis under many conditions and through different routes [13]. The rate of HIF-1α-positive cells in HCC tissues exceeds 94%, and the profile of the HIF-1α protein correlates with HCC cell differentiation and intrahepatic and extrahepatic metastases [14]. The STAT3/HIF-1α signaling pathway is of great importance in HCC. For example, HIF-1α and the IL-6-JAK-STAT3 signaling pathways were significantly activated and accelerated hepatocellular carcinoma growth after longer hepatic pedicle clamping followed by major hepatectomy [15]. A previous study found that TRIM14 activates the STAT3 pathway and induces tumor progression in melanoma [16]. Notwithstanding, we have no idea how the STAT3/HIF-1α signaling pathway is modulated by TRIM14 functions in HCC.

The study probes into the expression features of TRIM14 in HCC tissues and cells and its interplay with STAT3/HIF-1α to disclose the molecular mechanism associated with HCC cells. TRIM14 is considerably upregulated in HCC. Furthermore, TRIM14 overexpression initiates the STAT3/HIF-1α pathway to facilitate HCC cell proliferation, migration, and invasion and to trigger cell resistance to cisplatin. Our research may offer a novel therapeutic target for HCC treatment.

## 2. Results

### 2.1. TRIM14 Upregulation Was Correlated with Poorer Prognosis of HCC Patients

**Figure 1:**
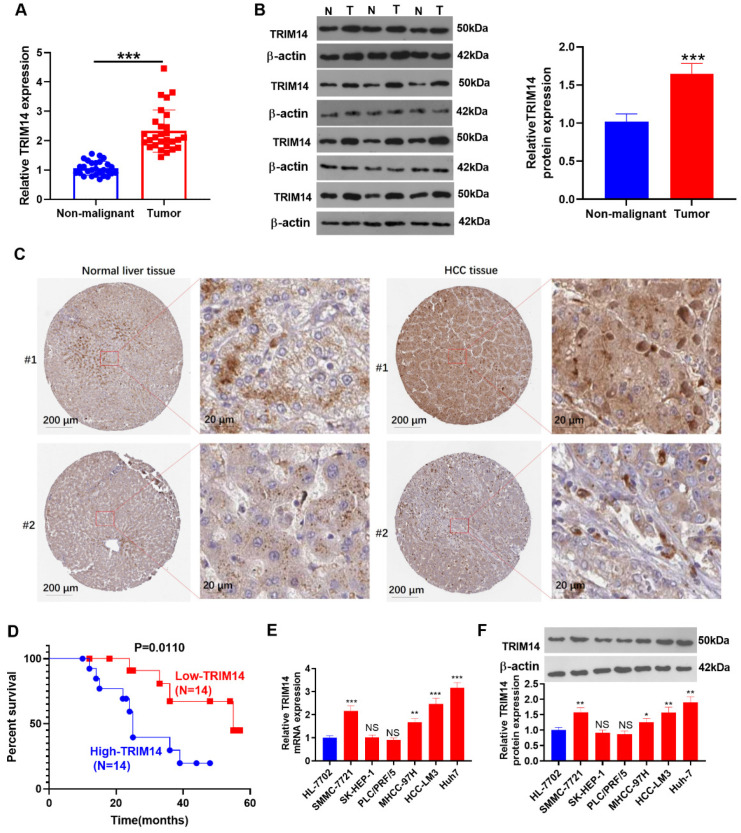
The association of TRIM14 upregulation with HCC patient prognosis. (**A**) qRT-PCR verified TRIM14 expression in HCC tissues (T) and adjacent nonmalignant tissues (N), n = 28. (**B**) Western blot verified TRIM14 expression in HCC tissues (T) and adjacent nonmalignant tissues (N), n = 12. (**C**) The IHC images of TRIM14 in HCC tissues and normal liver tissues were analyzed through the Human Protein Atlas database (https://www.proteinatlas.org/, accessed on 23 May 2021). (**D**) The survival curve of HCC patients with high or low levels of TRIM14. The median level of TRIM14 was a cut-off value. (**E**,**F**) TRIM14 expression in normal liver cells and HCC cells was confirmed with qRT-PCR (**E**) and Western blot (**F**). N = 3. NS *p* > 0.05, * *p* < 0.05, ** *p* < 0.01, and *** p < 0.001 (vs. the HL-7702 group).

qRT-PCR was performed to examine TRIM14 expression in 28 HCC tissues and adjacent nontumor tissues. Its expression was substantially increased in HCC tissues (*p* < 0.05, Figure 1A). Western blot analysis of TRIM14 expression in HCC tissues and nonmalignant tissues showed that TRIM14 expression was markedly upregulated in HCC tissues (*p* < 0.05, Figure 1B). The histopathological expression of TRIM14 in HCC tissues was analyzed through the Human Protein Atlas database (https://www.proteinatlas.org/, accessed on 23 May 2021). TRIM14 was expressed in both the cytoplasmic and membranous parts of normal liver cells and HCC tumor cells (Figure 1C). The Kaplan-Meier curve was used to analyze the correlation between *TRIM14* gene expression and the cumulative survival time of HCC patients, revealing that the overall survival rate of patients with a high TRIM14 expression was notably lower than that of patients with a low TRIM14 expression (*p* = 0.0110, Figure 1D). qRT-PCR and Western blotting revealed TRIM14 expression in HCC cell lines and normal liver cells (HL-7702[L-O2]), indicating that TRIM14 expression was remarkably elevated in HCC cells (SMMC-7721, MHCC-97H, HCC-LM3, and Huh-7) compared to normal liver cells (*p* < 0.05, Figure 1E,F). These outcomes revealed that a high TRIM14 expression correlated with the poor prognosis of HCC patients.

### 2.2. TRIM14 Overexpression Strengthened HCC Cell Proliferation and Metastasis

**Figure 2:**
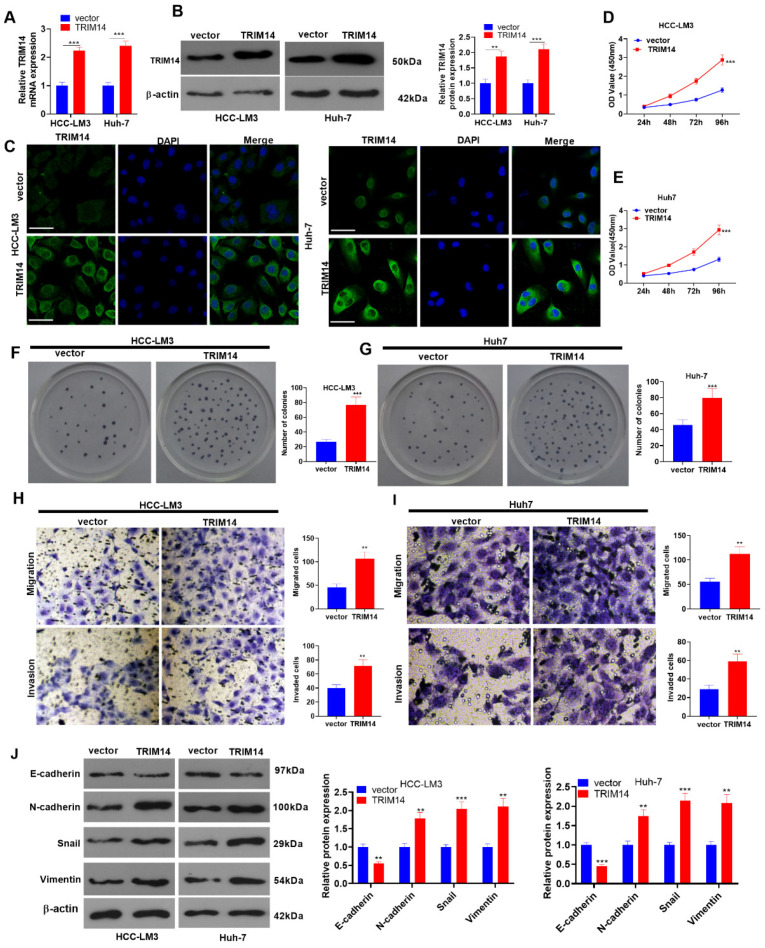
To determine the role of *TRIM14* overexpression in HCC cell proliferation and metastasis, HCC cells (HCC-LM3 and Huh-7) were transfected along with negative vector or TRIM14 overexpression plasmids. (**A**,**B**) TRIM14 expression in HCC cells was determined with qRT-PCR and Western blot. (**C**) Immunofluorescence was used to detect TRIM14 (green color) localization in HCC cells. Scale bar = 20 μm. The blue color shows DAPI. (**D**,**E**) CCK8 was used to examine HCC cell viability. (**F**,**G**) A clone formation assay was used to detect cell proliferation. (**H**,**I**) Transwell assays were used to monitor HCC cell migration and invasion. Magnification: 100×. (**J**) Western blot revealed the profiles of EMT-correlated proteins (including E-cadherin, N-cadherin, Snail, and Vimentin). ** *p* < 0.01 and *** *p* < 0.001 (vs. the vector group). N = 3.

To investigate the influence of TRIM14 on the malignant phenotype of HCC cells, we transfected HCC cells (HCC-LM3 and Huh-7) along with negative vector and TRIM14 overexpression plasmids. qRT-PCR, Western blot, and immunofluorescence verified the profile of TRIM14. In contrast with the vector group, the TRIM14 expression in cells was prominently augmented in the TRIM14 overexpression group (*p* < 0.05, Figure 2A–C). A CCK8 assay and clone formation assay were implemented to examine cell viability and proliferation, respectively. The data showed that TRIM14 overexpression contributed to a significant increase in the viability and proliferation of HCC cells compared to the vector group (*p* < 0.05, Figure 2D–G). Transwell assays were used to monitor cell migration and invasion, reflecting that in comparison with the vector group, TRIM14 overexpression led to a distinct increase in cell migration and invasion (*p* < 0.05, Figure 2H,I). Western blotting was carried out to detect the profiles of EMT-associated proteins (E-cadherin, N-cadherin, Snail, and Vimentin). When TRIM14 was overexpressed, the E-cadherin protein expression was conspicuously lowered in HCC cells, whereas the profiles of N-cadherin, Snail, and Vimentin were vastly elevated (*p* < 0.05, Figure 2J). Given these findings, overexpressed TRIM14 could increase HCC cell proliferation and metastasis.

### 2.3. TRIM14 Overexpression Enhanced HCC Cell Cisplatin Resistance and Autophagy

**Figure 3:**
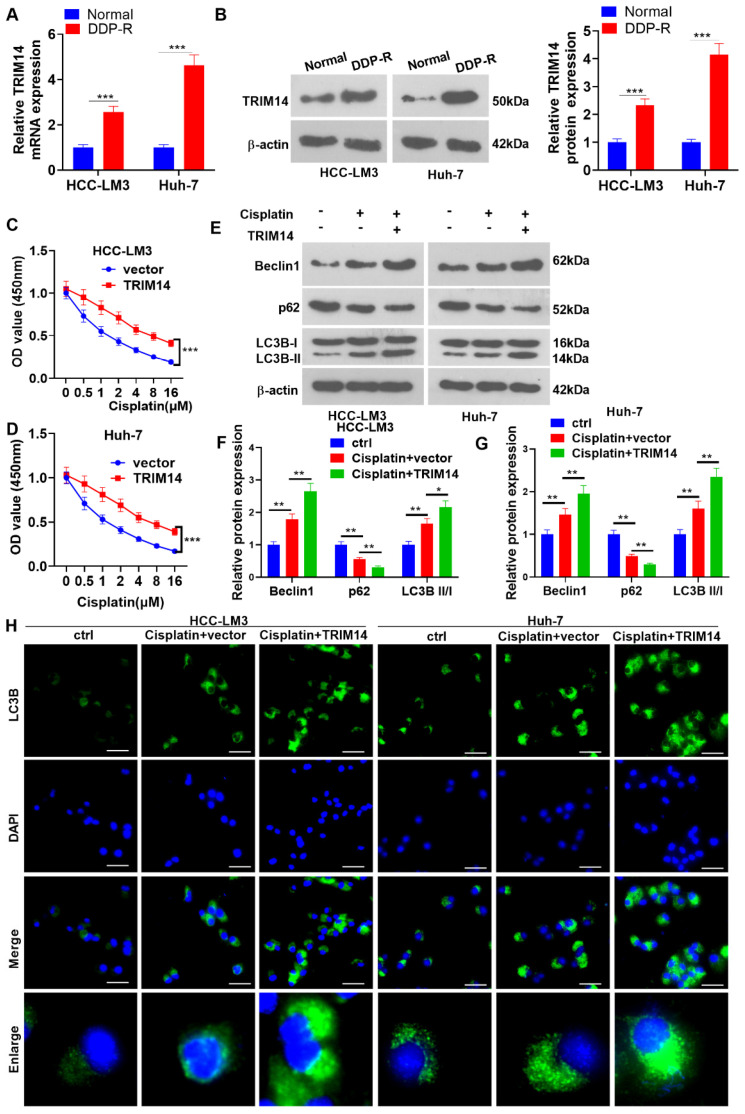
The role of TRIM14 overexpression in HCC cell cisplatin resistance and autophagy. (**A**,**B**) qRT-PCR and Western blot verified TRIM14 expression in cisplatin-resistant HCC cells (DDP-R). DDP at different concentrations (0, 0.5, 1, 2, 4, 8, and 16 μM) was applied to treat HCC cells that were transfected along with vector or TRIM14 overexpression plasmids. (**C**,**D**) CCK8 assay was used to evaluate the viability of HCC cells. (**E**–**G**) Western blot revealed the profiles of autophagy-correlated proteins (including LC3B, Beclin1, and p62). (**H**) The cells were treated with cisplatin (5 μM). Immunofluorescence was used to detect LC3B (green color) expression in the cells. Scale bars = 20 μm. The blue color shows DAPI. * *p* < 0.05, ** *p* < 0.01 and *** *p* < 0.001 (vs. the vector group). N = 3.

To understand the impact of TRIM14 on HCC cell resistance to cisplatin, we performed qRT-PCR and Western blotting to determine the TRIM14 expression in cisplatin (DDP)-resistant HCC cells (DDP-R). In contrast with the normal group, the TRIM14 level had an evident increase in the cisplatin-resistant cells (*p* < 0.05, Figure 3A,B). DDP at various concentrations (0, 0.5, 1, 2, 4, 8, and 16 μM) was applied to treat the cells for 24 h, with CCK8 assay adopted to examine their viability. In contrast to the vector group, the TRIM14 overexpression group experienced a dramatic increase in cell viability (*p* < 0.05, Figure 3C,D), suggesting that TRIM14 overexpression reduced the response of HCC cells to cisplatin. Next, cell autophagy was detected. The data showed that cisplatin treatment enhanced Beclin1 and LC3BII/I levels and reduced p62 expression. TRIM14 overexpression further elevated Beclin1 and LC3BII/I levels and reduced p62 expression (Figure 3E–G). Moreover, TRIM14 overexpression enhanced the accumulation of LC3 puncta (Figure 3H). These discoveries revealed that TRIM14 overexpression contributed to a lower response of HCC cells to cisplatin and strengthened autophagy.

### 2.4. TRIM14 Downregulation Inhibited HCC Cell Proliferation and Augmented Apoptosis

**Figure 4:**
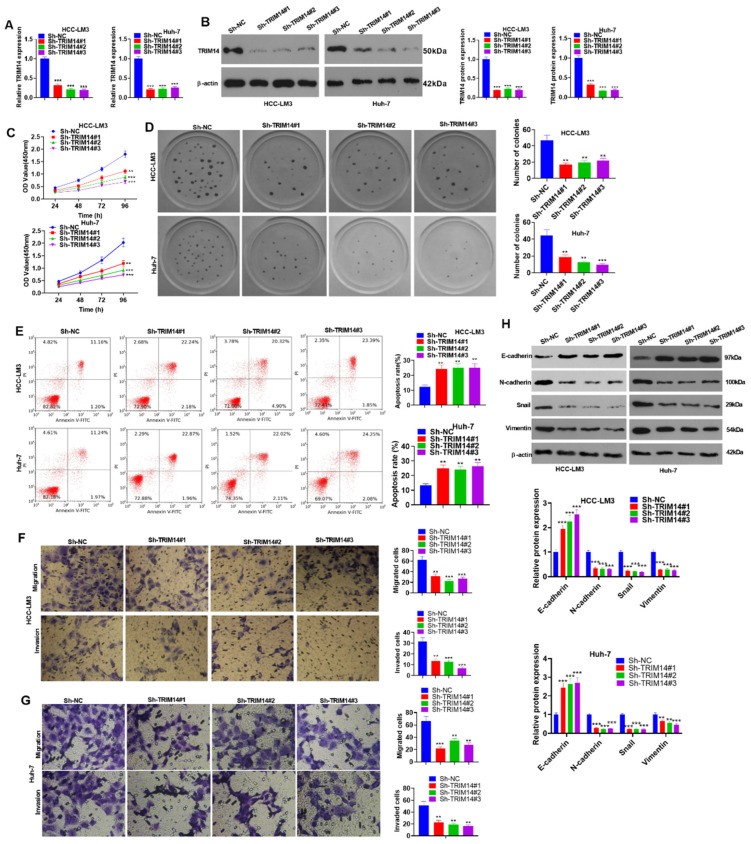
To determine the role of TRIM14 downregulation in HCC cell proliferation, apoptosis, and metastasis, HCC cells (HCC-LM3 and Huh-7) were transfected along with sh-NC or sh-TRIM14. (**A**,**B**) TRIM14 expression in HCC cells was determined with qRT-PCR and Western blot. (**C**) CCK8 was used to examine HCC cell viability. (**D**) A clone formation assay was used to detect cell proliferation. (**E**) Flow cytometry was used to track apoptosis. (**F**,**G**) Transwell assays were used to monitor HCC cell migration and invasion. Magnification: 100×. (**H**) Western blot revealed the profiles of EMT-correlated proteins (including E-cadherin, N-cadherin, Snail, and Vimentin). ** *p* < 0.01 and *** *p* < 0.001 (vs. the sh-NC group). N = 3.

To delve into the function of low TRIM14 expression in HCC cells, we transfected sh-NC, sh-TRIM14#1, sh-TRIM14#2, and sh-TRIM14#3 into HCC cells (HCC-LM3 and Huh-7). qRT-PCR and Western blot verified the profile of TRIM14, indicating that in contrast with Sh-NC, TRIM14 presented low expression in Sh-TRIM14 (#1, #2, and #3) (*p* < 0.05, Figure 4A,B). The CCK8 assay and clone formation assay were used to examine cell viability and proliferation, respectively, and the results indicated that when TRIM14 was knocked down, HCC cell proliferation was reduced (*p* < 0.05, Figure 4C,D). Flow cytometry measured cell apoptosis, signifying that TRIM14 knockdown greatly increased cell apoptosis (*p* < 0.05, Figure 4E). Transwell assays were used to monitor HCC cell migration and invasion and showed that TRIM14 knockdown substantially inhibited the migration and invasion of HCC cells (*p* < 0.05, Figure 4F,G). Western blot data suggested that TRIM14 knockdown gave rise to a conspicuous increase in E-cadherin expression, while the profiles of N-cadherin, Snail, and Vimentin were obviously reduced in HCC cells (*p* < 0.05, Figure 4H). These findings unveiled that TRIM14 inhibition could suppress HCC cell proliferation and metastasis and augment apoptosis.

### 2.5. TRIM14 Inhibition Enhanced HCC Cell Sensitivity to Cisplatin and Reduced Autophagy

**Figure 5:**
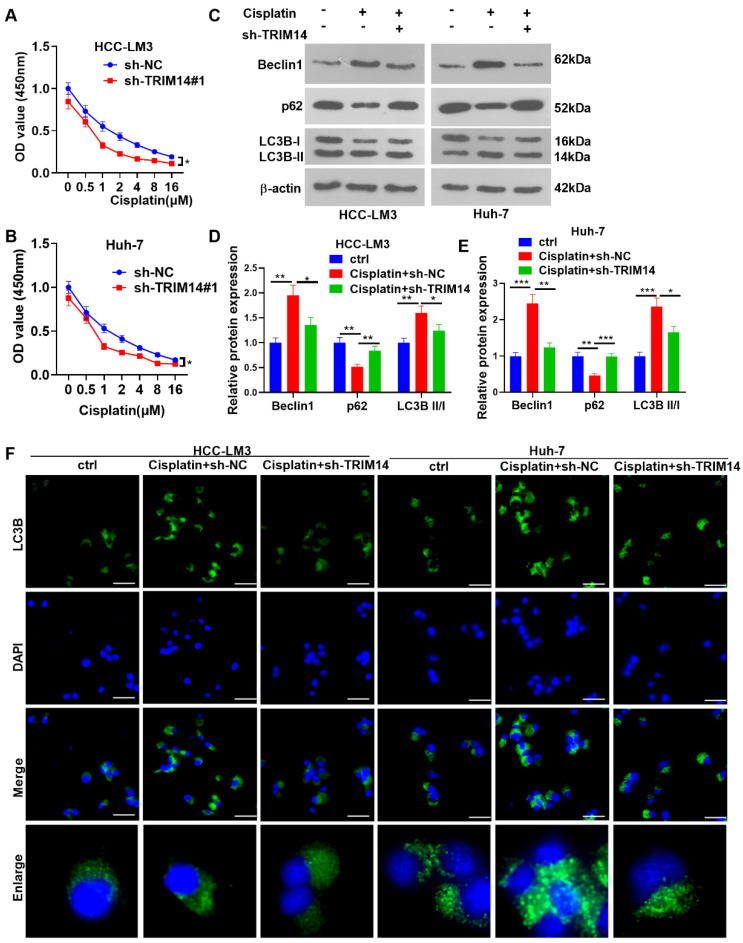
The role of TRIM14 downregulation in HCC cell cisplatin resistance and autophagy. DDP of different concentrations (0, 0.5, 1, 2, 4, 8, and 16 μM) was applied to treat HCC cells that were transfected along with sh-NC or sh-TRIM14. (**A**,**B**) CCK8 assay was used to evaluate the viability of HCC cells. (**C**–**E**) Western disclosed blot revealed the profiles of autophagy-correlated proteins (including LC3B, Beclin1, and p62). (**F**) The cells were treated with cisplatin (5 μM). Immunofluorescence was used to detect LC3B (green color) expression in the cells. Scale bars = 20 μm. The blue color shows DAPI. * *p* < 0.05, ** *p* < 0.01 and *** *p* < 0.001 (vs. the vector group). N = 3.

To probe into how TRIM14 inhibition influences the drug resistance of HCC cells, we tested the viability of HCC cells with TRIM14 knockdown. The CCK8 assay indicated that in contrast with the sh-NC group, TRIM14 knockdown remarkably hampered the viability of HCC cells treated with cisplatin (*p* < 0.05, Figure 5A,B). Cell autophagy was detected with Western blotting. The data showed that compared with the cisplatin + Sh-NC group, the TRIM14 downregulation reduced Beclin1 and LC3BII/I levels and promoted p62 expression (Figure 5C–E). Moreover, the TRIM14 downregulation mitigated the accumulation of LC3 puncta (Figure 5F). Therefore, knocking down TRIM14 and inhibiting the resistance of HCC cells to cisplatin and autophagy.

### 2.6. TRIM14 Overexpression Enhanced Tumor Growth In Vivo

**Figure 6:**
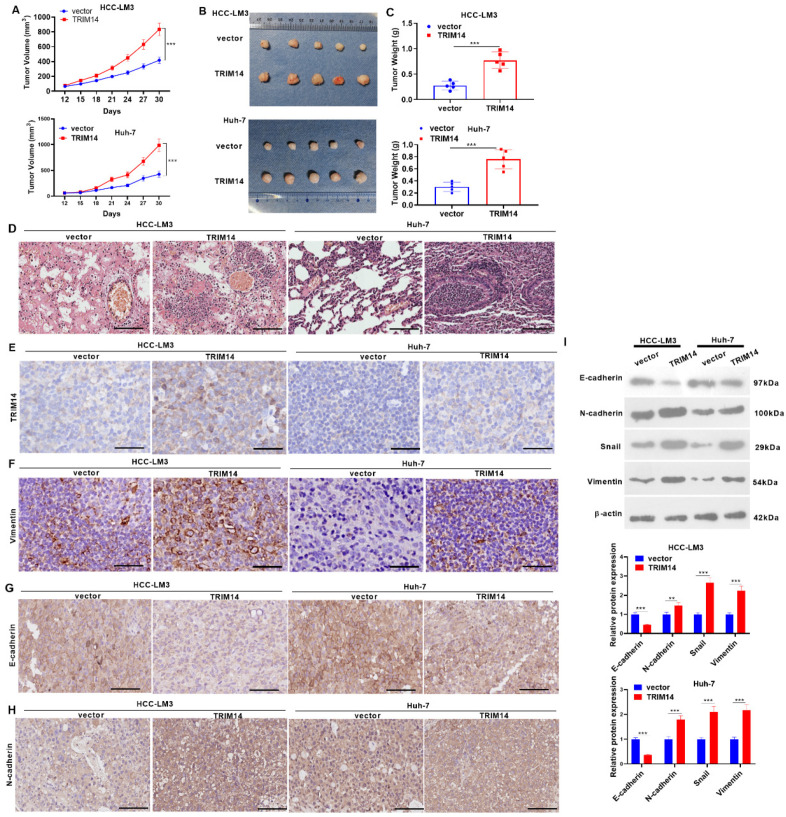
The role of TRIM14 overexpression in the growth and metastasis of HCC cells in vivo. HCC cells (HCC-LM3 and Huh-7) transfected with negative vector or TRIM14 overexpression plasmid were subcutaneously inoculated into nude mice. On Day 30 after treatment, the nude animals (n = 5 in each group) were killed via cervical dislocation. (**A**–**C**) The tumor volume (**A**), images (**B**), and weight (**C**). (**D**) The lung metastasis amount was determined through caudal vein injection, and HE staining was used to evaluate lung metastasis of tumor cells. (**E**–**H**) Immunohistochemistry confirmed TRIM14 (**E**), Vimentin (**F**), E-cadherin (**G**), and N-cadherin (**H**) expression. Scale bar = 100 μm. (**I**) Western blot analysis of the profiles of EMT-associated proteins (E-cadherin, N-cadherin, Snail, and Vimentin) in tumor tissues. ** *p* < 0.01 and *** *p* < 0.001 (vs. the vector/Si-NC group). N = 5.

To confirm the function of TRIM14 in HCC cell growth in vivo, we engineered a nude mouse tumor formation model and gauged the mouse tumor volume and mass. In comparison with the vector group, the tumor volume and mass of the mice overexpressing TRIM14 were apparently augmented (*p* < 0.05, Figure 6A–C). As displayed by the HE staining of the mouse lung tissues, in contrast to the vector group, TRIM14 overexpression enhanced the metastasis of the lung tissues (Figure 6D). As exhibited by immunohistochemistry, in contrast with the vector group, the TRIM14 overexpression group had a higher expression of TRIM14, Vimentin, and N-cadherin in the tumor, while E-cadherin was repressed following TRIM14 overexpression. In comparison with the Si-NC group, TRIM14 knockdown decreased the percentages of positive TRIM14 and Vimentin cells in the tumor cells (Figure 6E–H). Western blot verified the profiles of EMT-related proteins. In contrast with the vector group, TRIM14 overexpression greatly decreased E-cadherin expression and upregulated the profiles of N-cadherin, Snail, and Vimentin in the tumor tissues (*p* < 0.05, Figure 6I). TRIM14 overexpression facilitated the growth and metastasis of tumors in tissues.

### 2.7. TRIM14 Overexpression Activated the STAT3/HIF-1α Pathway in HCC Tissues and Cells

**Figure 7:**
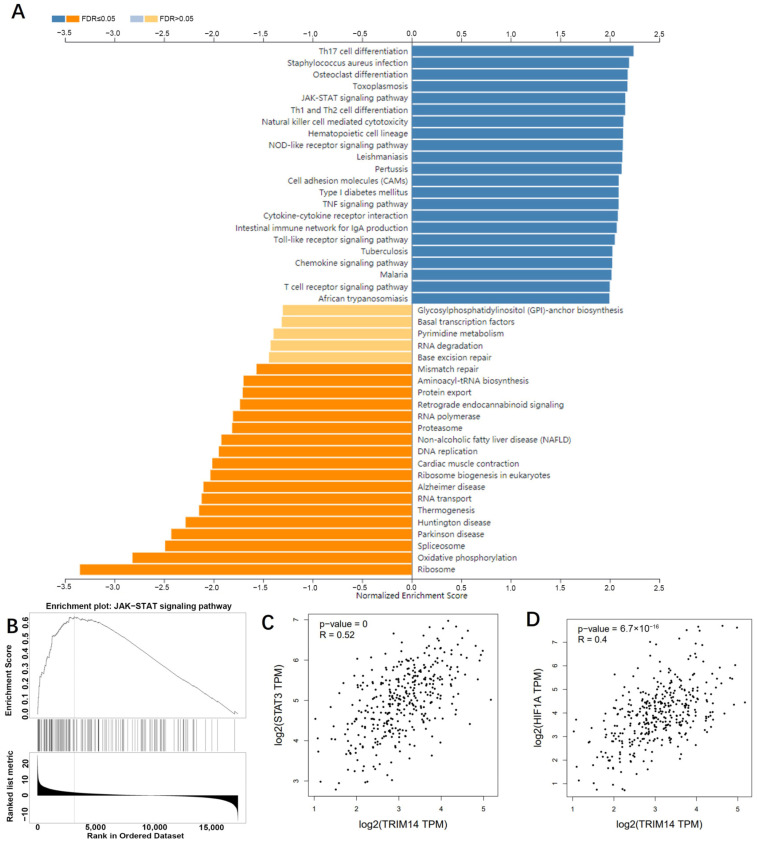
TRIM14 potentially activates the JAK-STAT pathway in HCC. (**A**) Gene function enrichment analysis was performed, and KEGG pathways that are associated with TRIM14 are shown. (**B**) The JAK−STAT signaling pathway was positively related to TRIM14. (**C**,**D**) The relationship of *STAT3* and *HIF-1A* with TRIM14 in LIHC was analyzed in GEPIA (http://gepia.cancer-pku.cn/, accessed on 25 May 2021).

**Figure 8:**
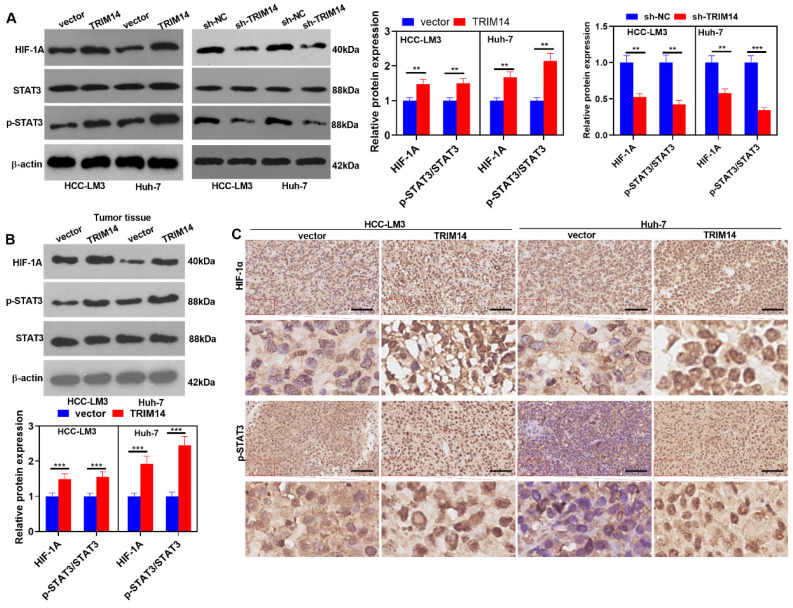
TRIM14 overexpression activated STAT3/HIF-1α expression in HCC tissues and cells. (**A**) Western blot revealed the profile of STAT3/HIF-1α in HCC cells where TRIM14 was overexpressed or knocked down. (**B**) STAT3/HIF-1α expression in the tumor tissues was verified with Western blot. (**C**) Immunohistochemistry confirmed HIF-1α and p-STAT3 expression. Scale bar = 100 μm. N = 3. ** *p* < 0.01 and *** *p* < 0.001 (vs. the vector or sh-NC group).

To dig deep into the mechanisms of TRIM14 in HCC, we analyzed the positive-related and negative-related genes of TRIM14 in liver hepatocellular carcinoma (LIHC) via LinkedOmics (http://linkedomics.org/login.php, accessed on 25 May 2021). KEGG gene function enrichment analysis using the online tool LinkedOmics indicated that the JAK-STAT signaling pathway was positively related to TRIM14 (Figure 7A,B). In LIHC tissues, *STAT3* and *HIF-1A* were positively related to TRIM14 (Figure 7C,D). We conducted Western blotting to verify STAT3/HIF-1α expression in HCC cells with a TRIM14 overexpression or knockdown. The phosphorylation of STAT3 was apparently strengthened, and the profile of HIF-1α was evidently heightened in the TRIM14 overexpression group compared with the vector group. In contrast to the Sh-NC group, the TRIM14 knockdown reduced STAT3 phosphorylation and dramatically lowered HIF-1α expression (Figure 8A). In the tumor tissues, STAT3 phosphorylation was strengthened, and *HIF-1A* expression was distinctly upregulated in the TRIM14 overexpression group compared to the vector group (*p* < 0.05, Figure 8B,C). These phenomena revealed that TRIM14 overexpression promoted STAT3/HIF-1α expression in HCC.

### 2.8. STAT3 or HIF-1α Inhibition Mitigated TRIM14-Mediated Malignancy

**Figure 9:**
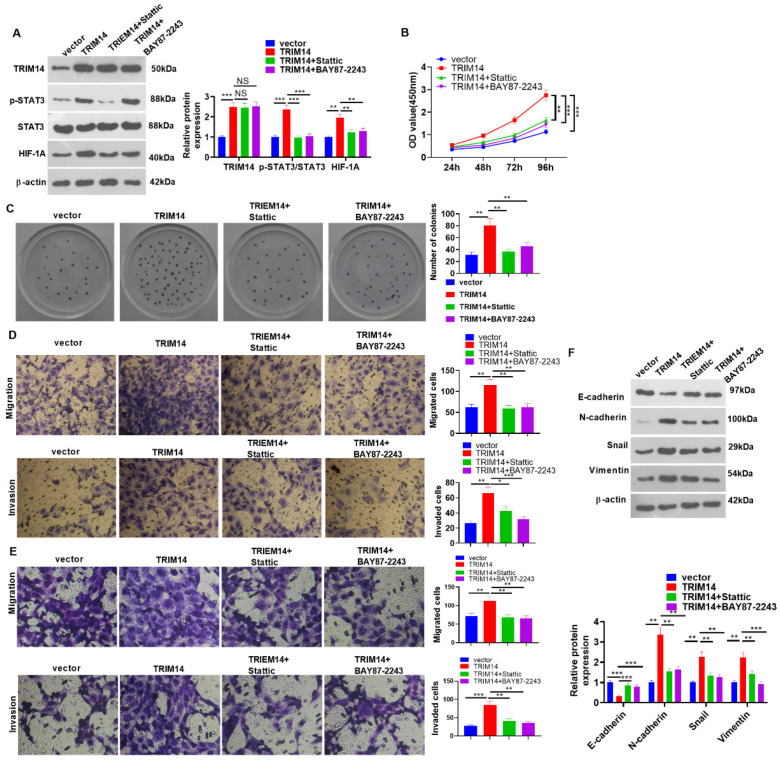
The effects of STAT3 or HIF-1α inhibition on HCC cell proliferation and metastasis and apoptosis. HCC cells (HCC-LM3 and Huh7) were transfected along with vector and TRIM14 overexpression plasmids, respectively. Following overexpression transfection, Stattic (the STAT3 inhibitor) or BAY87-2243 (the HIF-1A inhibitor) was added. (**A**) Western blot confirmed the protein profiles of TRIM14, STAT3, p-STAT3, and HIF-1α in HCC-LM3 cells. (**B**) CCK8 assay was used to examine HCC-LM3 cell viability. (**C**) A clone formation assay was used to detect cell proliferation. (**D**,**E**) Transwell assays were used to monitor cell migration and invasion of HCC-LM3 and Huh7 cells. Magnification: 100×. (**F**) The profiles of EMT-associated proteins (E-cadherin, N-cadherin, Snail, and Vimentin) in HCC-LM3 cells were unveiled with Western blot. NS *p* > 0.05, * *p* < 0.05, ** *p* < 0.01 and *** *p* < 0.001 (vs. the TRIM14 group). N = 3.

To corroborate the mechanisms of STAT3/HIF-1α and TRIM14 in HCC, we transfected TRIM14 overexpression plasmids into HCC cells (HCC-LM3). Subsequent to overexpression transfection, Stattic (the STAT3 inhibitor) and BAY87-2243 (the HIF-1α inhibitor) were added. Western blotting was used to ascertain the protein expression of TRIM14, p-STAT3/STAT3, and HIF-1α. Stattic and BAY87-2243 had no significant effect on TRIM14 expression. The p-STAT3 level was reduced by Stattic but not by BAY87-2243. HIF-1A levels were repressed by Stattic and BAY87-2243 (Figure 9A). The CCK8 assay and clone formation assay monitored HCC cell viability and proliferation, reflecting that in contrast with the TRIM14 group, cell viability and proliferation were considerably attenuated in the TRIM14+ Stattic group and the TRIM14+ BAY87-2243 group (*p* < 0.05, Figure 9B,C). Transwell assays were used to track cell migration and invasion of HCC-LM3 and Huh7 cells. Their migration and invasion both declined dramatically in the TRIM14+ Stattic group and the TRIM14+ BAY87-2243 group compared with the TRIM14 group (*p* < 0.05, Figure 9D,E). Western blot analysis of EMT-associated proteins was conducted. In contrast with the TRIM14 group, STAT3 or HIF-1α inhibition both boosted E-cadherin protein expression and restrained the profiles of N-cadherin, Snail, and Vimentin (*p* < 0.05, Figure 9F). In light of the above findings, STAT3 or HIF-1α inhibition mitigated the promoting effects of TRIM14 on the growth of HCC cells.

## 3. Discussion

Hepatocellular carcinoma, an exceedingly harmful malignancy with a high incidence in Asia, is prone to intrahepatic and extrahepatic metastasis, featuring an extremely poor prognosis. Moreover, Asian countries tend to use more aggressive interventions than European and American countries. Therefore, better prevention and treatment of HCC require exploration [17]. Here, we confirmed that TRIM14 affects the proliferation, sensitivity to chemotherapy drugs, apoptosis, migration, invasion, and autophagy of HCC cells. Moreover, TRIM14 activates STAT3 and HIF-1α pathways in HCC cells. Therefore, this study suggests that TRIM14 aggravates HCC development and chemoresistance. TRIM14 functions as an attractive therapeutic target for primary HCC as well as HCC with chemoresistance.

TRIM protein families pertain to the pathogenesis of multiple human cancers, taking part in the modulation of many cellular functions. For instance, TRIM14 expression is elevated in diverse cancers, such as gastric cancer and cervical cancer [18,19]. Overexpressed TRIM14 can bolster breast cancer cell proliferation, adding to the malignancy of colorectal cancer [20,21]. TRIM25 and TRIM29 overexpression can both enhance the malignant degree of gastric cancer [22,23]. TRIM11 and TRIM52 upregulation can boost HCC cell proliferation, migration, and invasion [24,25]. The findings resemble those of our research. Highly expressed TRIM14 has also been verified to correlate with poor HCC prognosis [26], which is aligned with our research. Here, we discovered that TRIM14 overexpression strengthened HCC malignancy, including promoting cell proliferation, migration, invasion, autophagy, and EMT, as well as reducing apoptosis.

Reduced apoptosis and enhanced autophagy are outstanding characteristics during the formation of chemoresistance in HCC cells [27,28,29]. Mediating apoptosis and autophagy by altering gene expression or drugs has been found to be effective in reversing cisplatin resistance. For example, mitochondrial fission factor (Mff), a protein that regulates the process of dividing mitochondria into smaller fragments (also known as mitochondrial fission), was promoted in cisplatin-resistant HCC. Mff knockdown enhances cell apoptosis and inhibits mitochondrial fission, thus sensitizing Huh-7/DDP cells to cisplatin treatment [30]. Astragaloside IV, a rich component from *Astragalus membranaceus*, reduced Cisplatin resistance by increasing tumor cell apoptosis and suppressing MRP2 expression [31]. In a hypoxic environment, enhanced autophagy was found to be related to sorafenib and cisplatin resistance in HCC cells. The autophagy inhibitor 3-MA markedly eliminated sorafenib and cisplatin resistance [32,33]. Presently, we observed that cisplatin promoted TRIM14 upregulation and autophagy. TRIM14 overexpression promoted autophagy and cisplatin resistance, while TRIM14 knockdown exerted the opposite effects. Previous studies have also indicated that TRIM14 is upregulated in 5-FU- and L-OHP-resistant gastric cancer tissues and cells. Functionally, TRIM14 overexpression promoted the proliferation and autophagy of SGC7901/5-FU cells and inhibited apoptosis [34]. Therefore, TRIM14 potentially mediates HCC development by affecting apoptosis and autophagy.

The STAT3/HIF-1α signaling pathway exerts a promoting function in various cancers. For instance, STAT3/HIF-1α signaling pathway activation augments laryngeal cancer cell resistance to cisplatin [9]. STAT3/HIF-1α signaling pathway inhibition can attenuate the resistance of breast cancer cells to adriamycin [35]. TRIM14 overexpression activates the AKT and STAT3 pathways to boost melanoma proliferation [16], which corresponds to our study. Here, we mainly probed the functions of TRIM14 and the STAT3/HIF-1α signaling pathway in HCC cells. Aberrant STAT3 expression has been previously shown to facilitate the malignant development of multiple human cancers. For example, excessive STAT3 activation can bolster osteosarcoma cell proliferation and strengthen cell resistance to adriamycin and cisplatin [36]. As displayed by our statistics, TRIM14 overexpression could enable STAT3 phosphorylation to initiate the STAT3/HIF-1α signaling pathway; considerably increase HCC cell proliferation, metastasis, and cisplatin resistance; and boost their apoptosis. In contrast, TRIM14 knockdown repressed the profile of STAT3/HIF-1α and increased HCC cell apoptosis. Moreover, we also uncovered that STAT3 or HIF-1α inhibition could mitigate the promoting function of TRIM14 overexpression in proliferation and metastasis. All the discoveries further demonstrated that highly expressed TRIM14 exerted its carcinogenic function in HCC by virtue of the STAT3/HIF-1α pathway.

Several shortcomings need further investigation in the future. First, the functions of TRIM14 should be confirmed in more HCC cell lines that have altered TRIM14 expression and primary HCC cells. Second, more clinical samples are required for verifying the diagnostic role of TRIM14 in HCC. Third, the role of TRIM14 knockdown in HCC development should be explored in vivo.

## 4. Materials and Methods

### 4.1. Specimen Collection

The clinical study was approved by the Ethics Committee of Shanghai Cancer Center of Fudan University (approval no. 050432-4-2108*) in 2021, and all patients signed written consent forms. A total of 28 HCC patients who had undergone hepatobiliary surgery at the hospital (16 males and 12 females) participated in the study. HCC tissue and adjacent para-carcinoma tissue samples harvested during surgery were quick-frozen immediately and stored at −80 °C. Immunohistochemistry, qRT-PCR, and Western blotting were used to determine the profiles of TRIM14 in tissues and cells.

### 4.2. Cell Culture

Human hepatoma cell lines (SMMC-7721, SK-HEP-1, BEL-7402, MHCC-97H, HCC-LM3, PLC/PRF/5, and Huh-7) and human normal liver cells (HL-7702 (L-O2)) were purchased from the American Type Culture Collection (ATCC, Rockville, MD, USA). Human HCC cells, seeded into complete RPMI1640 culture medium incorporating 10% inactivated newborn bovine serum (FBS, HyClone, Logan, UT, USA), were routinely cultivated with 5% CO_2_ and saturated humidity at 37 °C. The cells, passaged once every two or three days, were taken for experiments when they reached the logarithmic growth phase.

### 4.3. Cell Transfection

Lentiviral vectors expressing the TRIM14 gene or the short-hairpin RNA (shRNA) targeting TRIM14 (Sh-TRIM14#1, Sh-TRIM14#2, and Sh-TRIM14#3) and their negative controls (sh-NC and vector) were designed based on the open reading frame (ORF) region of TRIM14. Those expression vectors were synthesized by Guangzhou RiboBio Co., Ltd. (RiboBio, Guangzhou, China). These vectors were transfected into HCC-LM3 and Huh-7 cells using FuGENE HD Transfection Reagent (Roche, Darmstadt, Germany) as instructed by the manufacturer. Forty-eight hours following transfection, the total RNA of the cells was extracted, and TRIM14 mRNA level was assessed for confirming transfection efficiency. Seventy-two hours after transfection, total protein was extracted from the cells, and TRIM14 protein level was tested using Western blot for confirming transfection efficiency.

### 4.4. Quantitative Reverse Transcription PCR (qRT-PCR)

TRIzol reagent (Invitrogen, Waltham, MA, USA) was adopted to extract total RNA from human HCC tissues, mouse tumor tissues, and HCC cells per the supplier’s protocol. A Nanodrop spectrophotometer was manipulated to gauge the concentration and purity of the RNA. The PrimeScript-RT Kit (Madison, WI, USA) was employed to synthesize 1 µg of the total RNA into complementary DNA (cDNA), as stipulated by the manufacturer, followed by qRT-PCR with the help of SYBR^®^Premix-Ex-Taq™ (Takara, TX, USA) and the ABI7300 system. The total volume of the PCR system was set to 30 µL, and each sample encompassed 300 ng cDNA. The amplification procedure was implemented as follows: 10 min of initial denaturation at 95 °C, then 45 cycles covering 10 s at 95 °C, 30 s at 60 °C, and 20 s at 85 °C. All the fluorescence statistics were transformed into relative quantification, and β-actin was regarded as the internal parameter of TRIM14. qRT-PCRs were repeated three times using the following primers—TRIM14: forward 5’-AAGGCCCAGTACTCAAGGTC-3’, reverse 5’-ACTACTGGGAGGTTGACGTG-3’; β-actin: forward CCTGCTTGCTGATCCACATC, reverse CCTCTATGCCAACACAGTGC.

### 4.5. Western Blot

The collected cells were rinsed in cold PBS three times. Then, 100~200 μL of RIPA lysis buffer (Beyotime Biotechnology, Shanghai, China) was added to lyse the cells in ice-cold water under ultrasound, and the Bradford method was used to check the protein concentration. Proteins of identical amounts were taken from each group for 10% SDS-PAGE electrophoresis, and the proteins on the gel were transferred onto PVDF membranes (Millipore, Bedford, MA, USA). After being sealed for an hour at 4 °C, the membranes were incubated with anti-TRIM14 (15742-1-AP, 1:500, Proteintech, Wuhan, China), anti-STAT3 (ab68153, 1:1000, Abcam, Cambridge, UK), anti-p-STAT3 (phospho Y705, ab76315, Abcam, 1:1000), anti-HIF-1α (ab187524, Abcam, 1:1000), anti-p62 (ab109012, Abcam, 1:1000), anti-LC3B (ab63817, Abcam, 1:2000), anti-Beclin1 (ab207612, Abcam, 1:2000), anti-E-cadherin (ab76319, Abcam, 1:1000), anti-N-cadherin (ab76011, Abcam, 1:2000), anti-Snail (ab180714, Abcam, 1:1000), anti-Vimentin (ab92547, Abcam, 1:1000), and anti-β-actin (ab8226, 1:000, Abcam) overnight at 4 °C. Then, goat anti-rabbit IgG H&L (HRP) (ab97051, 1:1000) was administered for 1 h of hybridization at indoor temperature. The membranes were flushed with TBST three times for 5 min each. The ECL chemiluminescence substrate was used for color development, and X-ray films were exposed for 30 s to 5 min.

### 4.6. Cell Counting Kit-8 (CCK8) Assay

Cisplatin-resistant HCC cells (HCC-LM3/DDP and Huh-7/DDP) and HCC cells (HCC-LM3 and Huh-7) were in the logarithmic growth stage and digested with trypsin. Cell number was calculated using Countess™ Cell Counting Chamber Slides (Catalog number: C10228, Invitrogen™). With the cell density adjusted to 2 × 10^4^/mL, 100 μL medium was added to 96-well plates. Cisplatin at different concentrations was administered to treat the cells for 24 h, and the 96-well plates were further cultured in an incubator. A 10 μL CCK8 solution (Beyotime Biotechnology, Shanghai, China) was added to each well after 24, 48, or 96 h to incubate the plates for another 1 h in the incubator. As the culture ended, a microplate reader in which the 96-well plates were placed was exploited to check the absorbance (OD value) of each well at 450 nm, after which the determination was taken at the 24th, 48th, 72nd, and 96th hours.

### 4.7. Clone Formation Assay

HCC-LM3 and Huh-7 cells were seeded in 60 mm dishes at 1000 cells per dish. The cells were cultured in DMEM supplemented with 10% FBS, and the medium was exchanged with fresh medium every three days. After 10 days, the cell clones were rinsed two times with PBS, and 4% paraformaldehyde was used for cell fixation. Cell staining was performed using 0.05% crystal violet. After washing out the dye, the number of colonies (>50 cells/colony) was counted using light microscopy (Olympus, Tokyo, Japan).

### 4.8. Transwell Assay

*Trypsin* (0.25%) was employed to disperse HCC cell lines (HCC-LM3, Huh-7), which were then centrifuged, resuspended, and spread into the wells of a 24-well culture plate. Matrigel Chambers (8 µm pore size; Corning, Beijing, China) were harnessed in the invasion test instead of the migration assay. The upper chamber, coated with Matrigel beforehand, accommodated 5 × 10^4^ transfected cells. A medium with 10% FBS supplemented with 400 μL of RPMI-1640 was placed in the lower chamber. Following 24 h of incubation at 37 °C, the cells that failed to migrate were removed from the upper compartment. The Transwell membrane was immobilized with 4% paraformaldehyde for 10 min, dyed with 0.5% crystal violet, and then washed in double distilled water. An inverted microscope was adopted for counting. All trials were implemented in triplicate and repeated three times.

### 4.9. Flow Cytometry

Human HCC cells (HCC-LM3 and Huh-7) were made into a single-cell suspension, then inoculated into a culture flask (25 cm^2^). The original medium was discarded after the cells adhered to the wall overnight. A medium incorporating 0.3% FBS was added to the experimental group, while a medium containing PBS of a comparable volume was delivered to the control group. The media were cultivated in an incubator with 5% CO_2_ at 37 °C for 24 h, and the cell supernatant was collected. After three washes with cold PBS, trypsin with EDTA excluded was utilized to digest and collect the cells. The remaining steps were completed in line with the instructions of the Annexin V-PI Apoptosis Detection Kit (Yeasen Biotech Co., Ltd., Shanghai, China). Flow cytometry was performed within one hour, and apoptosis was analyzed.

### 4.10. Nude Mouse Tumor Formation Model

HCC cells (HCC-LM3 and Huh-7) were transfected along with vector or TRIM14 overexpression plasmids, then cultivated with RPMI 1640 supplemented with 10% fetal bovine serum in an incubator with 5% CO_2_ at 37 °C. Female nude mice on a BALB/c background, 4–6 weeks of age and 180–200 g in weight, were acquired from the animal experimental center of Fudan University. They were reared under specific pathogen-free (SPF) conditions. Under aseptic circumstances, 0.9% normal saline was used to transform HCC-LM3 and Huh-7 cells in the logarithmic growth stage into a single-cell suspension, with the cell concentration adjusted to 1 × 10^7^/mL. When the mice were narcotized with 0.1 mL of 10% chloral hydrate, 0.1 mL of the cell suspension was subcutaneously transfused into each mouse in the right armpit with the help of a 1 mL syringe, with 10 mice in each group. The mental conditions, diet, activities, and defecation of the nude animals were monitored. The mouse weight was gauged on the day of inoculation, after which the weight was measured every five days, and a Vernier caliper was adopted to examine the long and short diameters of the tumors. On Day 30 post treatment, the animals were killed through cervical dislocation. The weight and long and short diameters of the tumors were gauged, and the tumor volume was calculated (V).

The amount of lung metastases was determined through the caudal vein. Normal saline (0.9%) was employed to make HCC-LM3 and Huh-7 cell suspensions into single-cell suspensions, which were injected into the caudal vein of the mice, with the cell concentration adjusted to 5 × 10^6^/mL. Thirty days later, the nude mice transfused with HCC cells were killed, and their lung tissues were harvested for examination. The left lung tissues of the mice were immobilized with 4% paraformaldehyde, dehydrated, made transparent, waxed, embedded, and sectioned (5 μm in thickness). After HE staining and neutral gum sealing, a light microscope was utilized to track the staining outcomes.

### 4.11. Immunohistochemistry

The mouse tumor tissues, fixed with 10% formaldehyde, were embedded in paraffin. The obtained sections were then dewaxed and hydrated. Prior to immunohistochemical staining, the dewaxed sections were dried at 37 °C for 2 h, the endogenous peroxides were blocked with 1% H_2_O_2_ for 5 min, PBS was used for three washes, and immunostaining blocking solution was employed for 1 h of blocking. Next, the slices were incubated with anti-vimentin (Abcam, ab92547, 1:200), anti-HIF-1α (Abcam, ab, 1:200), anti-STAT3 (phospho Y705) (Abcam, ab267373, 1:200), and anti-TRIM14 (Proteintech, 15742-1-AP, 1:100) antibodies overnight at 4 °C. Following PBS washing, the sections were incubated together with antiserum linked to biotin at room temperature for an hour. After they were flushed again, 3,3-diaminobenzidine hydrochloride was used for 1 min coloring, double distilled water was used for rinsing, and hematoxylin was applied for 1 min dyeing.

### 4.12. Immunofluorescence

HCC cells (HCC-LM3 and Huh-7) were seeded in 24-well plates (2 × 10^5^ cells/well). Twenty-four hours later, the cells were fixed with 4% paraformaldehyde, permeabilized with 0.1% Triton X-100, and blocked with 5% goat serum at room temperature for 1 h. The cells were incubated with anti-LC3B antibody (ab48394, 1:200) or anti-TRIM14 (Proteintech, 15742-1-AP, 1:50) overnight at 4 °C. Following PBS washing, the cells were incubated with goat anti-rabbit IgG H&L (Alexa Fluor^®^ 488) (ab150077, 1:400) at room temperature for 2 h. The nuclei were stained with DAPI solution (Beyotime, Shanghai, China) at room temperature for 2 h. Finally, a fluorescence microscope (Olympus, Japan) was utilized to observe the staining images.

### 4.13. Statistical Analysis

The analysis was implemented with GraphPad Prism 8.0 software (GraphPad Software Inc., La Jolla, CA, USA), with the outcomes presented as the mean ± SD. An independent sample test was employed to analyze the statistical significance between groups, with *p* < 0.05 regarded as statistically meaningful. The correlation between TRIM14 mRNA expression and the cumulative survival time of HCC patients was examined with the Kaplan-Meier curve, with *p* < 0.05 considered statistically significant.

## 5. Conclusions

To summarize, our work has shown that TRIM14 is overexpressed in both HCC tissues and cells and that high TRIM14 expression correlates with poor prognosis in clinical HCC patients. The resistance of HCC cells to cisplatin is enhanced, resulting in malignant HCC progression. These findings further broaden our knowledge of HCC and corroborate that TRIM14 serves as an oncogenic gene in HCC. Thus, it may become a novel target for HCC treatment in the future.

## Data Availability

The data used to support the findings of this study are available from the corresponding author upon request.
